# Variants in congenital hypogonadotrophic hypogonadism genes identified in an Indonesian cohort of 46,XY under-virilised boys

**DOI:** 10.1186/s40246-017-0098-2

**Published:** 2017-02-16

**Authors:** Katie L. Ayers, Aurore Bouty, Gorjana Robevska, Jocelyn A. van den Bergen, Achmad Zulfa Juniarto, Nurin Aisyiyah Listyasari, Andrew H. Sinclair, Sultana M. H. Faradz

**Affiliations:** 1 Murdoch Childrens Research Institute, Melbourne, Victoria Australia; 2 Department of Paediatrics, University of Melbourne, Melbourne, Victoria Australia; 3 The Royal Children’s Hospital, Melbourne, Victoria Australia; 4 Division of Human Genetics, Centre for Biomedical Research, Faculty of Medicine, Diponegoro University (FMDU), JL. Prof. H. Soedarto, SH, Tembalang, Semarang, 50275 Central Java Indonesia

**Keywords:** Congenital hypogonadotrophic hypogonadism, Under-virilisation, Hypospadias, Targeted gene sequencing, Disorder of sex development

## Abstract

**Background:**

Congenital hypogonadotrophic hypogonadism (CHH) and Kallmann syndrome (KS) are caused by disruption to the hypothalamic-pituitary-gonadal (H-P-G) axis. In particular, reduced production, secretion or action of gonadotrophin-releasing hormone (GnRH) is often responsible. Various genes, many of which play a role in the development and function of the GnRH neurons, have been implicated in these disorders. Clinically, CHH and KS are heterogeneous; however, in 46,XY patients, they can be characterised by under-virilisation phenotypes such as cryptorchidism and micropenis or delayed puberty. In rare cases, hypospadias may also be present.

**Results:**

Here, we describe genetic mutational analysis of CHH genes in Indonesian 46,XY disorder of sex development patients with under-virilisation. We present 11 male patients with varying degrees of under-virilisation who have rare variants in known CHH genes. Interestingly, many of these patients had hypospadias.

**Conclusions:**

We postulate that variants in CHH genes, in particular *PROKR2*, *PROK2*, *WDR11* and *FGFR1* with *CHD7*, may contribute to under-virilisation phenotypes including hypospadias in Indonesia.

## Background

Proper function of the hypothalamic-pituitary-gonadal (H-P-G) axis is essential for the development of the reproductive system. Gonadotrophin-releasing hormone (GnRH), secreted by the hypothalamus, stimulates the biosynthesis and the release of gonadotrophins from the anterior pituitary gland. These gonadotrophins (luteinising hormone (LH) and follicle-stimulating hormone (FSH)) both play distinct roles in the gonads during embryonic development. In males, FSH stimulates the proliferation of immature Sertoli cells and spermatogonia [1]. FSH also stimulates the secretion of inhibin, which acts in a negative feedback loop directly to the anterior pituitary. LH stimulates the production and secretion of testosterone from the Leydig cells, which is thought to occur through the LH receptor after 10 weeks post conception [2]. Disruption to the H-P-G axis (through deficient production, secretion or action of the gonadotrophins) can result in hypogonadotrophic hypogonadism (HH). While this can be associated with additional anomalies or syndromes such as Dandy-Walker syndrome, Gorden Holmes syndrome and CHARGE [3], when observed alone, it is termed congenital or idiopathic HH (CHH) (OMIM 146110). CHH can be coupled with a decreased or absent sense of smell due to the abnormal migration of the GnRH neurons [4, 5]. The co-occurrence of CHH with anosmia is termed Kallmann syndrome (KS (OMIM 308700, 147950, 244200, 610628, 612370 and 612702)).

Estimates of the prevalence of CHH range between 1 and 10 in 100,000 live births, with approximately two thirds of cases arising from KS [6]. CHH in 46,XY males can cause a reduced level of circulating androgens due to hypogonadism. Isolated or apparently isolated CHH (i.e. in a patient with KS who does not complain of an absent or diminished sense of smell) is most commonly diagnosed in teenagers or young men who present with pubertal failure. During foetal development, testosterone is responsible for virilisation of the reproductive tract and dihydrotestosterone (DHT), a highly potent derivative of testosterone, drives differentiation of the external genitalia. The appearance of clinical characteristics depends on when HH begins. When GnRH deficiency occurs in the late foetal or early neonatal periods, a significant decrease in androgens can lead to some CHH patients being diagnosed postnatally with under-virilisation phenotypes such as cryptorchidism, micropenis [7] and, in some rare cases, hypospadias [8]. Patients also typically showed delayed or absent puberty including minimal virilisation, low libido, lack of sexual function and a reduced or absent growth spurt [3]. In addition to the physical anomalies, physiological impairments have also been reported such as low self-esteem, distorted body image and, in some cases, problems in sexual identity [9, 10]. Finally, CHH may be diagnosed following adolescence, later in life when infertility is a concern. Given these complex and significant physical and psychological implications, early diagnosis and treatment of CHH is essential. Clinically, CHH can phenocopy partial androgen insensitivity syndrome (PAIS) or other disorders of sex development (DSDs) in which a reduction of testosterone during development can cause reduced virilisation. If blood hormone testing is not routinely carried out, these patients may be misdiagnosed and clinical management may differ.

Genetically, CHH is highly complex. More than 30 genes have been implicated in CHH and/or KS including nine genes that cause an overlapping syndrome [3]. To complicate matters, a large degree of variability in inheritance, penetrance and expressivity is seen in CHH and an increasing body of evidence suggests that this disorder can be caused by variants in more than one gene (oligogenicity) [11, 12]. Variants in known CHH genes currently account for only 50% of CHH cases [13] meaning that more genes are yet to be found. Here, we present genetic mutational analysis of CHH genes in Indonesian 46,XY patients presenting with under-virilisation phenotypes.

## Materials and methods

### Clinical data

Patients with 46,XY DSD were referred to the Center for Biomedical Research, Faculty of Medicine, Diponegoro University (FMDU), Semarang, Indonesia. The medical ethics committee of the Dr. Kariadi Hospital/FMDU approved this study, and informed consent was obtained from all participants, as well as their parents or guardians, prior to their participation in this study. Following informed consent, a detailed interview was performed at recruitment and data concerning medical history, age of initial presentation, sex of rearing, family history (relatives with a genital disorder) and consanguinity were collected. Patients were clinically evaluated by a trained andrologist; a detailed description of the external genitalia was obtained and, in many cases, images taken. A blood sample was obtained for karyotyping, hormonal analysis and DNA extraction. Referral and data collection took place between 2004 and 2010. Eighty-eight of these patients have been described previously [14]. A total of 47 males with 46,XY under-virilisation phenotypes (including uni- or bilateral cryptorchidism, hypospadias, bifid scrotum, micropenis and, in some cases, severe hypospadias) were included in this study. Hormone analysis was carried out for some patients including base level LH and FSH and testosterone (*T*). Reference levels for FSH and LH are based on paediatric measurements depending on age [15]. In some cases, *T* levels were also measured following Leydig cell stimulation by human chorionic gonadotrophin (hCG). For more details on blood hormone analysis, see [14].

### Gene panel sequencing

Genomic DNA was obtained from peripheral EDTA-blood samples using the salting out method [16]. The DNA underwent quality control at the Murdoch Childrens Research Institute (MCRI), Melbourne, Australia. Total genomic DNA was sequenced using a targeted panel (Haloplex, Agilent) that covers 64 diagnostic DSD genes [17]. This included 19 genes implicated in CHH (*CHD7*, *GNRH1*, *GNRHR*, *HESX1*, *LEP*, *PROKR2*, *PROP1*, *TAC3*, *FGFR1*, *KAL1*, *LHX3*, *FGF8*, *PROK2*, *KISS1R*, *WDR11*, *SPRY4*, *FSHB*, *CGA*, *SOX10*). Library preparation and sequencing were carried out as detailed in [17]. Raw data was analysed using a modified pipeline created at MCRI—C-pipe, which calls variants and provides data on frequency and pathogenicity [18].

Following C-pipe analysis, variants were checked for quality and depth and were filtered for those less than 1% minor allele frequency (MAF) in both the ESP6500 and 1000 genome project. As non-affected controls from Indonesia were not included, variants that were found very frequently in our screen (greater than 5% of total samples run) were also discounted. We manually check variant frequency in EVS and extracted ExAC data on frequency in Asia (South Asia and East Asia). Variants were checked for previous implication in human disease via ClinVAR and HMGD. Predicted pathogenicity of each variant was analysed using a range of up-to-date in silico prediction tools (SIFT, PolyPhen-2, LRT and MutationTaster). Effects on protein structure and function were predicted using the HOPE tool [19].

## Results

### Patient cohort

**Fig. 1:**
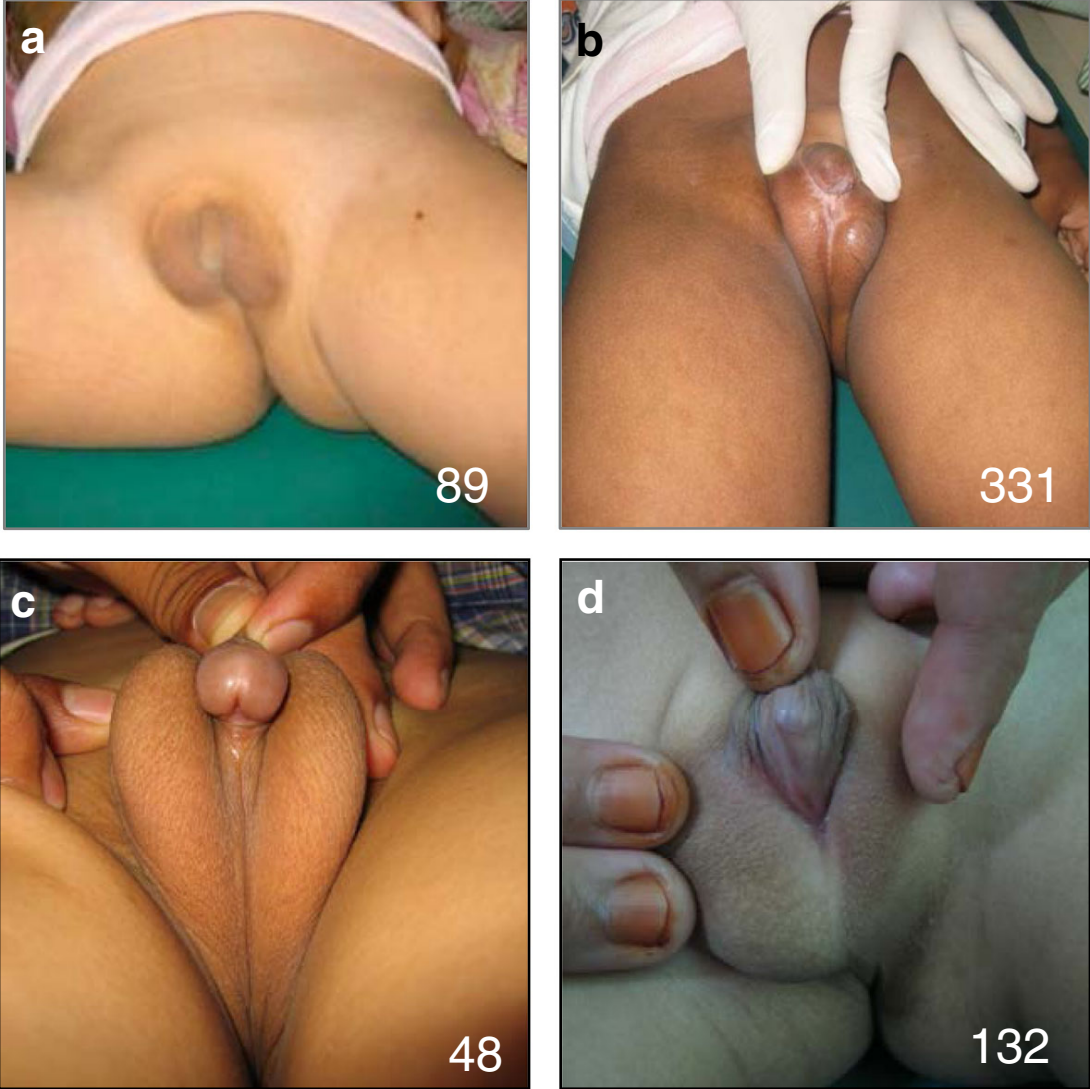
Under-virilisation in patients with CHH gene variants. **a**–**d** Representative images of external genitalia for four patients presenting with 46,XY DSD (see Table 1 for details)

All forty-seven 46,XY DSD patients from Indonesia were first analysed for mutations in DSD genes that cause androgen insensitivity or reduced testosterone production (e.g. *androgen receptor* (﻿*AR*), *SRD5A2*, *HSD17B3*). Rare and damaging mutations in these genes were found in 19 patients [17] of the 47. The other 28 did not have a causative variant identified. These patients ranged in age from newborn to 14 years old, with a variety of 46,XY DSD phenotypes including hypospadias, bifid scrotum, cryptorchidism/undescended testis, microtestis and micropenis. All patients identify as male. Many have undergone hypospadias repair. The phenotypes of the eleven patients with a CHH variant are shown in Table 1, and representative images are shown in Fig. 1.

**Table 1:** Patient clinical details

Patient ID	Age at initial appointment	Gender		Clinical description				Associated malformations	Anosmia reported?	hCG stimulation test	Image provided?
		Genetic	Sex of rearing	Testes	Scrotum	Micropenis	Urethral meatus (type of hypospadias)			Increased *T*?	
173	12	46,XY	Male	Bilaterally non palpable	Bifid	Yes	Scrotal	Spina bifida	Unknown	Moderate	
143	6	46,XY	Male	R, not palpable L, 1 ml, scrotal	Bifid	Yes	Scrotal		No		Figure 1b
159	2	46,XY	Male	R, 1 ml, scrotal L, fetractile	Bifid	No	Perineal		Unknown		
171	4	46,XY	Male	R, 1–2 ml, scrotal L, 2 ml, scrotal	Bifid	Yes	Scrotal		No	Yes	Figure 1a
47	3	46,XY	Female, changed to male at 3 years	R, 2 ml, scrotal L, not palpable	Bifid	Yes	Scrotal		No	Yes	
174	3	46,XY	Male	R, not palpable L, 1 ml, scrotal	Fused	Yes	Penoscrotal		No	Yes	
164	3	46,XY	Male	Bilaterally 2 ml, scrotal	Bifid	No	Penoscrotal		No	Yes	
163	10	46,XY	Male	Bilaterally 3 ml, scrotal	Bifid	Yes	Penile		No	Yes	Figure 1c
147	1 m	46,XY	Male	R, inguinal L, not palpable	Bifid	No	Scrotal		No	Yes	
101	3	46,XY	Male	Bilaterally 2 ml, scrotal	Bifid	Yes	Scrotal		Unknown	Yes	Figure 1d
169	14	46,XY	Male	R, 4 ml, scrotal	Bifid	no	Penoscrotal		No	Yes	
				L, 6 ml, scrotal							

Patient identification number and age at first consultation are shown, as well as sex chromosome complement and gender. A description of anomalies is also included. Response to hCG stimulation is shown. Testosterone reference levels were considered as 0.3–0.5 nmol/l except for patients 169, 163 and 8 (where reference was considered 3–6.5 nmol/l)

The hallmarks of CHH can include low levels of testosterone (due to hypogonadism), which can often be increased by hCG stimulation. Indeed, we found that all of the patients tested showed moderate to high increases in testosterone after hCG stimulation (Table 1). In addition, low levels of LH and FSH are often indicative of CHH; however, the natural levels of these hormones are low during childhood. Indeed, most patients were between mini-puberty and puberty when FSH/LH levels are expected to be low (<0.1–4 IU/l for LH and <0.1–8 IU/l for FSH). For all patients of this age, assayed LH and FSH levels were within the normal range. Patient 169, who was 14 at the time, had an LH measurement of 2.7 IU/l, which is within normal range, but an FSH of 9.24 IU/I, which is considered slightly elevated. Patient 147 was within mini-puberty at the time of measurement and subsequently had an elevated LH level of 10.8 and FSH of 6.23. This may suggest that secretion of these gonadotrophins is not inhibited in this patient. Patient 143 did not have hormonal analysis.

### CHH genetic variants

The remaining 28 patients were then analysed for mutations in the exonic regions of CHH genes as previously detailed in [17]. Eleven patients had one or more rare variants (<1% MAF in g1000 and ESP6500) in a CHH gene (Table 2). In total, we found 14 variants in CHH genes in these patients. The variants are described below.

**Table 2:** Rare variants found in CHH genes in 46,XY DSD patients

Patient ID	CHH gene	Variant location	Change	Variant details	dbSNP	EVS MAF	ExAC total freq.	ExAC SA/EA	ClinVar/HGMD	In silico predictions	GERP++ RS score	Previous functional studies
173	PROKR2	chr20:5283278-5283279	G/A	PROKR2:NM_144773:c.C563T:p.S188L	rs376239580	0.0077	0.00002	0/0	Yes—likely pathogenic for CHH	3 of 4	5.31	Cole et al. (2008); Zhu et al. (2015)
143	PROKR2	chr20:5283278-5283279	G/A	PROKR2:NM_144773:c.C563T:p.S188L	rs376239580	0.0077	0.00002	0/0	Yes—likely pathogenic for CHH	3 of 4	5.31	Cole et al. (2008); Zhu et al. (2015)
159	PROKR2	chr20:5282850-5282851	C/T	PROKR2:NM_144773:c.G991A:p.V331M	rs117106081	0.0154	0.00652	0.03119/0.02901	Yes—CHH	0 of 4	2.01	Dodé (2006); Monnier et al. (2009); Cole et al. (2008)
171	PROKR2	chr20:5282787-5282788	A/C	PROKR2:NM_144773:c.T1054G:p.W352G	Not found	0	0.00000	0.00	Not found	4 of 4	5.05	
47	PROK2	chr3:71834136-71834137	C/T	PROK2:NM_001126128:c.G68A:p.R23H	Not found	0	0.00000	0/0	Not found	0 of 4	2.47	
174	WDR11	chr10:122650293-122650294	G/T	WDR11:NM_018117:c.G2409T:p.W803C	Not found	0	0.00000	0/0	Not found	4 of 4	5.79	
164	WDR11	chr10 :122630739-122630740	A/G	WDR11:NM_018117:c.A1352G:p.H451R	rs199920020	0	0.00007	0/0.00104	Not found	2 of 4	3.575	
163	WDR11	chr10:122626666-122626667	T/A	WDR11:NM_018117:c.T1279A:p.L427I	Not found	0	0.00000	0/0	Not found	3 of 4	3.11	
147	FGFR1	chr8:38287238-38287239	G/A	FGFR1:NM_001174063:c.C320T:p.S107L	rs140382957	0.0077	0.00253	0.0002393/0.0454	1 record—benign	2 of 4	3.6	Sato (2004); Sykiotis (2010); Fukami et al. (2013)
101	CHD7	chr8:61655556-61655557	G/T	CHD7: NM_017780:c.G1565T:p.G522V	rs142962579	0	0.00232	0.0003717/0.03098	Not found	3 of 4	5.67	
	FGFR1	chr8:38287238-38287239	G/A	FGFR1:NM_001174063:c.C320T:p.S107L	rs140382957	0.0077	0.00253	0.0002393/0.0454	1 record—benign	2 of 4	3.6	Sato (2004), Sykiotis (2010); Fukami et al (2013)
169	FGFR1	chr8:38287238-38287239	G/A	FGFR1:NM_001174063:c.C320T:p.S107L	rs140382957	0.0077	0.00253	0.0002393/0.0454	1 record—benign	2 of 4	3.6	Sato (2004); Sykiotis (2010); Fukami et al. (2013)
	CHD7	chr8:61713055-61713056	C/T	CHD7:NM_017780:c.C2347T:p.P783S	rs373873996	0	0.00009	6.152e−05/0.00117	1 record—benign for CHARGE	2 of 4	5.81	
	LEP	chr7:127892124-127892125	A/G	LEP:NM_000230:c.A53G:p.Y18C	rs148407750	0.0461	0.00041	6.056e−05/0.003004	Not found	0 of 4	1.13	

Patient number is shown and the gene, variant location and DNA change. The allele frequency (from ExAC) is shown for all populations (MAF) and also specifically for both South Asia (AS) and East Asia (EA). Details are shown in the variant in found in Clinvar or in HMGD, and if reported previously, the reference is shown. Four in silico prediction programs were used for each variant, and the number of these showing a likely pathogenic/damaging score is shown. GERP++ scores are also shown

#### PROKR2

Four patients had variants in the *PROKR2* gene. Two patients (173 and 143) had the same variant—*PROKR2*:c.C563T:pS188L (Table 2). This variant has not been found in our DSD panel previously (in over 300 DSD patients; see [17]) but has a total allele frequency of 1.65e−05 in ExAC (although it has not been recorded in SA or EA). This change has been recorded to be likely pathogenic (ClinVar) [20, 21] (Table 2). Previous functional analysis has shown this variant has a strong defect in G-protein coupling [21]. The two patients with this variant (patients 173, 143) had under-virilisation phenotypes including micropenis, scrotal hypospadias and cryptorchidism (Fig. 1, Table 1). Interestingly, one of these patients also had additional anomalies. Patient 173 had spina bifida, incontinence and suspected intellectual disability—suggesting additional genetic or environmental contributors (Table 1). Indeed, the mother of this patient had a suspected folic acid deficiency during pregnancy.

**Fig. 2:**
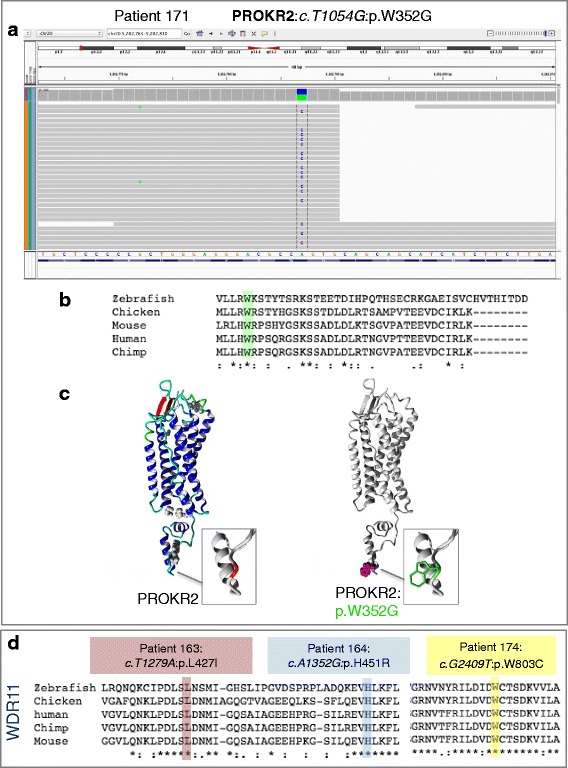
Novel variants in CHH genes. Just one novel variant in *PROKR2* was found (p.W352G). This change found in patient 171, *c.T1054G*:p.W352G, is heterozygous and has good quality and depth (**a**). This change falls on a highly conserved residue (**b**) and lies within the cytoplasmic tail of this transmembrane receptor (**c**). Three novel variants in WDR11 were found in our cohort—all of which affect a highly conserved residue (**d**)

Two other patients had heterozygous missense variants in the *PROKR2* gene (Table 2). c.G991A:p.V331M was found in patient 159 who has perineal hypospadias and unilateral cryptorchidism (Fig. 1, Table 1). This variant (rs117106081) has a total frequency in ExAC of 0.0065 (and was greater than 0.01 in both SA and EA). It is not predicted to be damaging in any of the in silico prediction tools and was not highly conserved. Nevertheless, this variant has been previously reported in CHH/KS patients, and functional analysis in both publications suggested a reduction in function (in particular a mild G-protein coupling defect) [21–23]. In contrast, another variant c.T1054G:p.W352G was not found in ExAC or EVS and was predicted to be damaging and highly conserved among different species (Table 2 and Fig. 2a). This was found in a patient 171, who has bilateral cryptorchidism and scrotal hypospadias (Fig. 1 and Table 1). In this case, the mutated residue is located on the surface of a domain with unknown function (Fig. 2b). The mutant residue (glycine) is smaller than the wild-type residue and differs in hydrophobicity to the wild-type residue (tryptophan). This may cause a loss of external interactions in particular a loss of hydrophobic interactions with other molecules on the surface of the protein.

From this, we hypothesise that *PROKR2* variants, in particular the variant p.S188L, represent a significant cause of under-virilisation including cryptorchidism, micropenis and, in some cases, hypospadias in Indonesian 46,XY DSD patients.

#### PROK2

One patient (47) was found to harbour a variant in this gene (*PROK2*:c.G68A:p.R23H). This missense heterozygous variant was not found in any of the online databases; however, it was not predicted to be pathogenic (Table 2). This patient has a micropenis, scrotal hypospadias and unilateral cryptorchidism (Table 1). The first 27 amino acids of PROK2 are a signal peptide, important for its secretion. The affected amino acid (arginine at position 23) lies within this region.

#### WDR11

Three patients had heterozygous missense variants in *WDR11* (Table 2, patients 174, 164, 163). The first of these was one of a pair of twins, who have concordant phenotypes (patient 174, twin not analysed). This variant, *WDR11*:c.G2409T:p.W803C, was not found in online databases and is predicted to be pathogenic with strong conservation—even down to zebrafish (Table 2, Fig. 2c). Patient 174 has a micropenis with penoscrotal hypospadias and chordee (Fig. 1, Table 1). The second variant found was *WDR11*:c.A1352G:p.H451R (Table 2). This variant (rs199920020) has a total frequency of 0.0001 in ExAC but is rare in Asia and was found in a patient with penoscrotal hypospadias and bifid scrotum but no micropenis or cryptorchidism (patient 164, Fig. 1, Table 1). Like the previous variant, this amino acid is highly conserved (Fig. 2c). The third variant (*WDR11*:c.T1279A:p.L427I) was not previously found in any online databases and was predicted to be damaging and highly conserved (Fig. 2c). This was found in patient 163, who has a micropenis and penile hypospadias (Fig. 1, Table 1).

#### FGFR1

Three patients had the *FGFR1* variant rs140382957 (*FGFR1*:c.C320T:p.S107L) (Table 2). This variant has a MAF in EVS of 0.0077 and in ExAC of 0.0023 (and while it is predicted to be pathogenic in two prediction tools, one record in ClinVar has it logged as being benign [24].)

#### CHD7

Curiously, two of the three patients who had a *FGFR1* variant also had a variant in *CHD7*. One of these, *CHD7*: c.G1565T:p.G522V has an ExAC MAF of 0.002318 and was predicted damaging (Table 2); however, it has been reported as benign for CHARGE on ClinVar. This was found in patient 101. Another novel variant was rare (ExAC MAF = 9.2e−05) (*CHD7*:c.C2347T:p.P783S). No other *CHD7* variants were found.

## Discussion

In this study, we have investigated variants in CHH-related genes in a cohort of Indonesian 46,XY DSD patients who had an under-virilisation phenotype. After excluding patients with mutations in known DSD genes, we found rare and damaging variants in CHH genes in 11 of the remaining 28 patients. CHH and KS can present at birth with under-virilisation phenotypes in males such as micropenis and cryptorchidism [25]. Our study suggests that CHH may be a cause of under-virilisation in Indonesia. While forty-seven 46,XY DSD patients were initially recruited, 19 of these were found to harbour mutation in a known DSD genes such as *AR* (data not shown). Of the remaining 28, we found a likely CHH variant(s) in 11 patients, making this a total of 25% of the total original cohort. In addition, while our targeted DSD panel has a comprehensive list of diagnostic DSD genes, it only covers 19 of approximately 24 genes that cause CHH/KS without an associated syndrome. Sequencing of the entire list of known CHH genes, including those that cause CHH in association with additional anomalies, may increase the diagnostic yield of a genetic screen like this. This will be important for future studies of this cohort.

Penile and urethral morphology is established before 14 weeks gestation meaning that the foetal pituitary-hypothalamic axis is typically thought to be unnecessary for normal penile development (instead relying on maternal hCG). However, after week 14, continued increase in penile length is dependent upon the hypothalamic-pituitary axis. Therefore, boys with hypogonadism will often have micropenis but normal phallic morphology. However, we found that many of individuals in our cohort had varying degrees of hypospadias. Given this, it is interesting to note that while rare, hypospadias in patients with CHH or KS has been described. A large study found two patients with CHH and hypospadias [26], and several other studies have described patients with KS or CHH and hypospadias of varying degrees [8, 27–29]. Nevertheless, hypospadias in CHH is a rare combination, and it is interesting to speculate why our cohort has an over-representation of variants in CHH genes in patients with hypospadias. It is possible that in our cohort of Indonesian patients, CHH and KS manifest in a unique way, as we have not found this association in patients with 46,XY DSD of other nationalities (data not shown). Or, it may be that these variants simply contribute to a phenotype in these patients that could involve additional undetected variants in genes controlling either gonadal or penile development. It is also possible that these genes/variants have an interaction with environmental cues in this population, resulting in more common under-virilisation in CHH than in other populations. Indeed, many of the described patients come from low socio-economic communities, and many of them are involved in agriculture. Both genetic and environmental factors are thought to contribute to isolated hypospadias (reviewed in [30]), and numerous studies in different populations have shown agriculture and pesticides to be a risk factor for reproductive development and health, e.g. [31–34]. Finally, it is possible that these variants detected in CHH genes are non-damaging variants over-represented in the Indonesian population. However, three patients had a *PROKR2* variant previously shown to be deleterious in functional studies, and we have sequenced more than 100 individuals from Indonesia (include severe DSD patients, parents and siblings) who were not enriched for these or other rare variants in CHH genes (data not shown).

Mutations in *PROK2* and *PROKR2* are thought to contribute to around 9% of patients with KS [23]. In our cohort, a total of four patients had a variant in *PROKR2* and one with *PROK2*. We also had three patients with *WDR11* variants, meaning this gene may also play a significant role in Indonesian 46,XY DSD patients. Overall, we have found eight variants in CHH genes that have not been previously described in this disorder. Of these, four are not present in online variant databases ExAC or EVS. The PROKR2 variant p.W352G lies within the cytoplasmic tail of this transmembrane receptor. Other variants have been described in this region (such as p.V331M—which we also found, and p.R357W) [21]. In this case, the mutant residue (glycine) is smaller than the wild-type residue and differs in hydrophobicity to the wild-type residue (tryptophan). This may cause a loss of external interactions in particular a loss of hydrophobic interactions with other molecules on the surface of the protein. One patient had a variant in *PROK2 (*p.R23H) that has not been previously described. The affected amino acid lies within the signal peptide region, and the mutant residue (histidine) is smaller than the wild-type residue and has a different charge (neutral rather than positive). This may change the activity of the signal peptide, and a patient with a variant affecting the neighboring amino acid (p.A24P) has been described previously in CHH [21].

The PROKR2 p.V331M variant that we and others have found has been shown to have reduced functional activity (albeit weaker than other variants) [21–23]; however, it is not predicted to be damaging by any of the four prediction tools used. This is likely due to the fact that several orthologous proteins in other species have a methionine in this protein position. It has been suggested that filtering variants based on currently available pathogenicity tools may lead to under-reporting of such compensated variants [35]. Therefore, while we have included the *in silico* predictions of pathogenicity in our pipeline, we have chosen to report all rare variants in this manuscript regardless of these predictions.

Finally, two novel *WDR11* variants were found in our screen. WDR11 is predicted to exhibit two β propellers made up of WD domains. Protein structure modelling has predicted that WDR11 has 12 WD domains and that nine of them (second through tenth) participate in the genesis of two consecutive β propellers [36]. The p.W803C variant in which a tryptophan is replaced by a cysteine at position 803 falls within the 12th WD domain and is a highly conserved amino acid [36]. Cysteine is a smaller residue than the wild-type residue, which could interrupt with the WD function. The p.L427I change is predicted to fall adjacent to WD domain 6, where at least two other human variants have been described [36].

Interestingly, we also found two patients with both *FGFR1* and *CHD7* variants. Indeed, oligogenicity has been described to be a feature of CHH (for a summary, see [3]). Specifically, oligogenic inheritance has been previously reported for *FGFR1*, while no reports for *CHD7* oligogenicity have yet been published. While *CHD7* has most frequently been associated with CHARGE syndrome, of which hypospadias can be a feature, a recent paper has detailed patients in which *CHD7* single-nucleotide variants (SNVs) were not associated with classical CHARGE syndromic features. Indeed, they show that rare deleterious SNVs in this gene contribute to the mutational burden of patients with both KS and CHH in the absence of full CHARGE syndromic features [37]. It may be that a combination of variant alleles in *FGFR1* and *CHD7* can cause hypospadias and under-virilisation. However, several of the *FGFR1* and *CHD7* variants had a total MAF of around 0.2%, with a prevalence of 0.3 or 0.4% in East Asia indicating that they may be over-represented in the Indonesian population. Further studies to address the pathogenicity of these variants and the interaction between *FGFR1* and *CHD7* are required and are beyond the scope of this study.

Hormonal analysis at the right age can be highly informative in a clinical diagnosis of CHH. This includes assays of the levels of the gonadotrophins FSH and LH, as well as testosterone levels before and after hCG stimulation. Diagnosis of KS and CHH in many of these patients has been limited by access to detailed blood hormone analysis (in particular as many are pre-pubescent children meaning that measuring LH and FSH is not informative). Most of our patients showed low levels of testosterone (consistent with their age), but these levels were stimulated by hCG. Nevertheless, the genetic results of this study suggest that boys presenting with under-virilisation phenotypes in Indonesian clinics should be tested for CHH or KS. The patients presented here will be monitored as they develop, and we recommend they have their gonadotrophin levels retested at a later date when reduced levels can be detected.

A genetic diagnosis can inform family planning and fertility investigations, as well as direct clinical management. Treatments exist for many of the features of CHH. In early life, this can include low-dose testosterone or gonadotrophins for micropenis and stimulation of gonadal development. Later, during adolescence or adulthood, testosterone therapy can also induce puberty including psychosocial development [3]. CHH-associated infertility can also be treated, for example, by administering GnRH or gonadotrophins [3]. Thus, given the therapeutic options, having a genetic diagnosis may allow earlier or tailored intervention. Gene panel testing is a viable option to deliver this genetic diagnosis.

## Conclusion

We conclude that variants in CHH genes, in particular *PROKR2*, *PROK2*, *WDR11* and *FGFR1* with *CHD7*, may contribute to under-virilisation phenotypes including hypospadias in Indonesian boys. We suggest that in this population, 46,XY DSD patients should be monitored for signs of CHH including hormonal and genetic analysis.

## Abbreviations

CHH: Congenital hypogonadotrophic hypogonadism
DHT: Dihydrotestosterone
DSD: Disorder of sex development
FSH: Follicle-stimulating hormone
GnRH: Gonadotrophin-releasing hormone
hCG: Human chorionic gonadotrophin
KS: Kallmann syndrome
LH: Luteinising hormone

## References

[1] Walker WH, Cheng J (2005). FSH and testosterone signaling in Sertoli cells. Reproduction.

[2] Svechnikov K, Landreh L, Weisser J, Izzo G, Colón E, Svechnikova I (2010). Origin, development and regulation of human Leydig cells. Horm Res Paediatr.

[3] Boehm U, Bouloux P-M, Dattani MT, de Roux N, Dodé C, Dunkel L (2015). Expert consensus document: European Consensus Statement on congenital hypogonadotropic hypogonadism—pathogenesis, diagnosis and treatment. Nat Rev Endocrinol.

[4] Teixeira L, Guimiot F, Dodé C, Fallet-Bianco C, Millar RP, Delezoide A-L (2010). Defective migration of neuroendocrine GnRH cells in human arrhinencephalic conditions. The Journal of clinical investigation. Am Soc Clin Invest.

[5] Schwanzel-Fukuda M, Pfaff DW (1989). Origin of luteinizing hormone-releasing hormone neurons. Nature.

[6] Bianco SDC, Kaiser UB (2009). The genetic and molecular basis of idiopathic hypogonadotropic hypogonadism. Nat Rev Endocrinol.

[7] Fraietta R, Zylberstejn DS, Esteves SC. Hypogonadotropic hypogonadism revisited. Clinics (Sao Paulo). Hospital das Clinicas da Faculdade de Medicina da Universidade de Sao Paulo. 2013;68 Suppl 1:81–8. 10.6061/clinics/2013(Sup01)09 PMC3583156 23503957

[8] Moriya K, Mitsui T, Tanaka H, Nakamura M, Nonomura K (2010). Long-term outcome of pituitary-gonadal axis and gonadal growth in patients with hypospadias at puberty. J Urol.

[9] Ediati A, Juniarto AZ, Birnie E, Drop SLS, Faradz SMH, Dessens AB (2013). Body image and sexuality in Indonesian adults with a disorder of sex development (DSD). J Sex Res.

[10] Ediati A, Faradz SMH, Juniarto AZ, van der Ende J, Drop SLS, Dessens AB (2015). Emotional and behavioral problems in late-identified Indonesian patients with disorders of sex development. J Psychosom Res.

[11] Izumi Y, Suzuki E, Kanzaki S, Yatsuga S, Kinjo S, Igarashi M (2014). Genome-wide copy number analysis and systematic mutation screening in 58 patients with hypogonadotropic hypogonadism. Fertil Steril.

[12] Raivio T, Sidis Y, Plummer L, Chen H, Ma J, Mukherjee A (2009). Impaired fibroblast growth factor receptor 1 signaling as a cause of normosmic idiopathic hypogonadotropic hypogonadism. J Clin Endocrinol Metab.

[13] Miraoui H, Dwyer AA, Sykiotis GP, Plummer L, Chung W, Feng B (2013). Mutations in FGF17, IL17RD, DUSP6, SPRY4, and FLRT3 are identified in individuals with congenital hypogonadotropic hypogonadism. Am J Hum Genet.

[14] Juniarto Z, van der Zwan YG, Santosa A, Ariani MD, Eggers S, Hersmus R, et al. Hormonal evaluation in relation to phenotype and genotype in 286 patients with a disorder of sex development from Indonesia. Clin Endocrinol (Oxf). 2016;n/a–n/a. 10.1111/cen.13051 26935236

[15] Soldin OP, Hoffman EG, Waring MA, Soldin SJ (2005). Pediatric reference intervals for FSH, LH, estradiol, T3, free T3, cortisol, and growth hormone on the DPC IMMULITE 1000. Clin Chim Acta.

[16] Miller SA, Dykes DD, Polesky HF (1988). A simple salting out procedure for extracting DNA from human nucleated cells. Nucleic Acids Res.

[17] Eggers S, Sadedin S, van den Bergen JA, Robevska G, Ohnesorg T, Hewitt J (2016). Disorders of sex development: insights from targeted gene sequencing of a large international patient cohort. Genome Biol.

[18] Sadedin SP, Dashnow H, James PA, Bahlo M, Bauer DC, Lonie A (2015). Cpipe: a shared variant detection pipeline designed for diagnostic settings. Genome Med.

[19] Venselaar H, Beek Te TAH, Kuipers RKP, Hekkelman ML, Vriend G (2010). Protein structure analysis of mutations causing inheritable diseases. An e-Science approach with life scientist friendly interfaces. BMC Bioinformatics.

[20] Zhu J, Choa RE-Y, Guo MH, Plummer L, Buck C, Palmert MR (2015). A shared genetic basis for self-limited delayed puberty and idiopathic hypogonadotropic hypogonadism. J Clin Endocrinol Metab.

[21] Cole LW, Sidis Y, Zhang C, Quinton R, Plummer L, Pignatelli D (2008). Mutations in prokineticin 2 and prokineticin receptor 2 genes in human gonadotrophin-releasing hormone deficiency: molecular genetics and clinical spectrum. J Clin Endocrinol Metab.

[22] Monnier C, Dodé C, Fabre L, Teixeira L, Labesse G, Pin J-P (2009). PROKR2 missense mutations associated with Kallmann syndrome impair receptor signalling activity. Hum Mol Genet.

[23] Dodé C, Rondard P (2013). PROK2/PROKR2 signaling and Kallmann syndrome. Front Endocrinol (Lausanne).

[24] Fukami M, Iso M, Sato N, Igarashi M, Seo M, Kazukawa I (2013). Submicroscopic deletion involving the fibroblast growth factor receptor 1 gene in a patient with combined pituitary hormone deficiency. Endocr J.

[25] Costa-Barbosa FA, Balasubramanian R, Keefe KW, Shaw ND, Tassan Al N, Plummer L (2013). Prioritizing genetic testing in patients with Kallmann syndrome using clinical phenotypes. J Clin Endocrinol Metab.

[26] Vizeneux A, Hilfiger A, Bouligand J, Pouillot M, Brailly-Tabard S, Bashamboo A (2013). Congenital hypogonadotropic hypogonadism during childhood: presentation and genetic analyses in 46 boys. Veitia RA, editor. PLoS ONE.

[27] Kurzrock EA, Delair S (2006). Hypospadias and Kallmann’s syndrome: distinction between morphogenesis and growth of the male phallus. J Pediatr Urol.

[28] Knorr JR, Ragland RL, Brown RS, Gelber N (1993). Kallmann syndrome: MR findings. AJNR Am J Neuroradiol.

[29] Ponticelli C, Frosini P, Masi L (1991). Kallmann’s syndrome. Apropos of 2 personal cases. Acta Otorhinolaryngol Ital.

[30] Bouty A, Ayers KL, Pask A, Heloury Y, Sinclair AH (2015). The genetic and environmental factors underlying hypospadias. Sex Dev.

[31] Strazzullo M, Matarazzo MR. Epigenetic effects of environmental chemicals on reproductive biology. Curr Drug Targets. 2016. 10.2174/1389450117666161025100125 27784215

[32] Bianca S, Li Volti G, Caruso-Nicoletti M, Ettore G, Barone P, Lupo L (2003). Elevated incidence of hypospadias in two sicilian towns where exposure to industrial and agricultural pollutants is high. Reprod Toxicol.

[33] Xu L-F, Liang C-Z, Lipianskaya J, Chen X-G, Fan S, Zhang L (2014). Risk factors for hypospadias in China. Asian J Androl.

[34] Kristensen P, Irgens LM, Andersen A, Bye AS, Sundheim L (1997). Birth defects among offspring of Norwegian farmers, 1967-1991. Epidemiology.

[35] Azevedo L, Mort M, Costa AC, Silva RM, Quelhas D, Amorim A (2016). Improving the in silico assessment of pathogenicity for compensated variants. Eur J Hum Genet.

[36] Kim H-G, Ahn J-W, Kurth I, Ullmann R, Kim H-T, Kulharya A (2010). WDR11, a WD protein that interacts with transcription factor EMX1, is mutated in idiopathic hypogonadotropic hypogonadism and Kallmann syndrome. Am J Hum Genet.

[37] Balasubramanian R, Choi J-H, Francescatto L, Willer J, Horton ER, Asimacopoulos EP (2014). Functionally compromised CHD7 alleles in patients with isolated GnRH deficiency. Proceedings of the National Academy of Sciences. National Acad Sci.

